# Outcomes of out-of-hospital cardiac arrest in Ireland 2012-2020: Protocol for an observational study

**DOI:** 10.12688/hrbopenres.13699.2

**Published:** 2023-10-31

**Authors:** Tomás Barry, Alice Kasemiire, Martin Quinn, Conor Deasy, Gerard Bury, Siobhan Masterson, Ricardo Segurado, Andrew Murphy, Out-of-Hospital Cardiac Arrest Registry Steering Group

**Affiliations:** 1 UCD School of Medicine, University College Dublin, Dublin, Leinster, D04 V1W8, Ireland; 2 UCD Centre for Support and Training in Analysis and Research, School of Public Health, Physiotherapy and Sports Science, University College Dublin, Dublin, Leinster, D04 V1W8, Ireland; 3 Out-of-Hospital Cardiac Arrest Register, National Ambulance Service, Donegal, D24 XNP2, Ireland; 4 School of Medicine, University College Cork, Cork, County Cork, T12 CY82, Ireland; 5 National Ambulance Service, Health Services Executive, Dublin, D24 XNP2, Ireland; 6 Discipline of General Practice, University of Galway, Galway, County Galway, H91 TK33, Ireland

**Keywords:** out-of-hospital cardiac arrest, registry data, observational research, prehospital emergency care, resuscitation, emergency medical services

## Abstract

**Background:**

Out-of-hospital cardiac arrest (OHCA) is a leading cause of preventable mortality that now affects almost 3,000 people each year in Ireland. Survival is low at 6–7%, compared to a European average of 8%. The Irish Out-of-Hospital Cardiac Registry (OHCAR) prospectively gathers data on all OHCA in Ireland where emergency medical services attempted resuscitation.

The Irish health system has undergone several developments that are relevant to OHCA care in the period 2012–2020. OHCAR data provides a means of exploring temporal trends in OHCA incidence, care, and outcomes over time. It also provides a means of exploring whether system developments were associated with a change in key outcomes.

This research aims to summarise key trends in available OHCAR data from the period 2012 – 2020, to explore and model predictors of bystander CPR, bystander defibrillation, and survival, and to explore the hypothesis that significant system level temporal developments were associated with improvements in these outcomes.

**Methods:**

The following protocol sets out the relevant background and research approach for an observational study that will address the above aims. Key trends in available OHCAR data (2012 – 2020) will be described and evaluated using descriptive summaries and graphical displays. Multivariable logistic regression will be used to model predictors of ‘bystander CPR’, ‘bystander defibrillation’ and ‘survival to hospital discharge’ and to explore the effects (if any) of system level developments in 2015/2016 and the COVID-19 pandemic (2020) on these outcomes.

**Discussion:**

The findings of this research will be used to understand temporal trends in the care processes and outcomes for OHCA in Ireland over the period 2012-2020. The results can further be used to optimise future health system developments for OHCA in both Ireland and internationally.

## Introduction

### Out-of-Hospital Cardiac Arrest

Out-of-hospital cardiac arrest (OHCA) describes the sudden loss of mechanical function of the heart and the absence of blood flow around the body, occurring outside of a hospital setting ^1, 2^. OHCA is the most time-critical medical emergency, with survival depending on the prompt actions of the community where OHCA occurs, emergency medical services (EMS) and in turn, appropriate follow-on hospital care ^3^. Internationally, the annual incidence of OHCA treated by emergency medical services (EMS) is estimated to be 30 – 97 individuals per 100,000 population, median age ranges from 64 to 79 years and more than half of victims are male ^4^. OHCA with attempted resuscitation has an incidence of 56 per 100,000 population per year in Europe and is considered the third leading cause of death ^5^. Survival to/ immediately after hospital discharge ranges from three to twenty percent internationally and is eight percent in Europe ^6^. Of those that do survive to discharge in Europe and North America, approximately seventy percent are still alive at three years ^7^. In Ireland there are now almost 3,000 OHCAs with attempted resuscitation each year of which six to seven percent involve survival to hospital discharge ^8^.

### The Chain of Survival

The ‘chain of survival’ describes the critical actions that can link an OHCA victim with survival ^9^. This chain involves early access to care, early cardiopulmonary resuscitation (CPR), early defibrillation and post resuscitation care ^10^. CPR and defibrillation are exquisitely time sensitive interventions and thus health systems face a significant challenge in ensuring OHCA patients have timely access to these treatments ^11^. Thankfully, the simplicity, affordability and increasing awareness of these interventions mean that they can be cascaded to the community and performed by ‘bystanders’ when cardiac arrest occurs ^12^. Thus, at population level these represent the most important OHCA interventions ^13, 14^. In circumstances where bystanders have not initiated CPR, dispatch assisted CPR (where the EMS emergency call taker provides CPR instruction to bystanders over the phone) can further improve survival following OHCA ^15^. 

CPR and defibrillation exist alongside several other OHCA interventions that can be delivered in the community and enroute to hospital by EMS. Randomised controlled trials have demonstrated that delivery of medications such as adrenaline and amiodarone may have additional benefits for some patient groups, while trials of airway management and mechanical CPR devices have failed to show significant survival improvements ^16– 19^. An additional novel but resource intense hospital (or hospital outreach) treatment ‘eCPR’ (extracorporeal cardiopulmonary resuscitation) uses a mechanical device to temporarily replace heart and lung function. eCPR has shown significant promise in specific OHCA sub-populations ^20^. However, the degree to which this treatment can be made available in an equitable fashion at population level is yet uncertain ^21, 22^. In terms of follow on in-hospital care, international guidelines now recommend that OHCA patients are cared for in specialist cardiac arrest centres, while acknowledging limited evidence for this recommendation outside of specific patient subgroups ^22, 23^.

### Out of Hospital Cardiac Arrest & Quality Improvement Registries

The science underpinning the ‘chain of survival’ is unquestionably of critical importance in OHCA care. However, scientific research alone will not be sufficient to improve OHCA outcomes and must be accompanied by a focus on real world implementation and quality improvement ^24^. To this end the European Resuscitation Council recommend that ‘health systems should have population-based registries which monitor the incidence, case-mix, treatment and outcomes for cardiac arrest’ and that these data ‘should inform health system planning and responses to cardiac arrest’ ^25^.

An internationally agreed ‘Utstein’ registry dataset has been devised which facilitates national and international comparison of OHCA ^26^. A recent pan European survey reported that six countries (including Ireland) had an OHCA registry with full population coverage, fourteen had partial population coverage and seven countries reported not having an OHCA registry ^27^. Comprehensive data on the Irish experience of OHCA is provided by the Irish Out-of-Hospital Cardiac Arrest Registry (OHCAR). OHCAR achieved national coverage using the Utstein dataset in 2012, and by 2020 this contains over 20,000 cases. In terms of the period considered in this study OHCAR does not include OHCA with EMS response but without attempted resuscitation. We anticipate that approximately fifty percent of cases recorded in OHCAR are witnessed (European average 66%), and twenty percent have an initial ‘shockable rhythm’ (European average 20%) ^6^. The primary sources of OHCAR data are patient care records and dispatch data from Ireland’s statutory ambulance services. OHCAR has provided invaluable continuous quality improvement data on an annual basis to ambulance services. To date however, no temporal analyses have been conducted to assess the impact of national interventions on survival in Ireland. Nor has the database been interrogated to determine whether international trends in OHCA (including increased bystander CPR and associated survival improvements ^28– 30^) are mirrored in the Irish data.

### Emergency medical services in Ireland

Ireland’s population is now in excess of five million people, for whom emergency medical services (EMS) are provided by the National Ambulance Service (NAS) ^31^. In Ireland’s capital Dublin, Dublin Fire Brigade (DFB) are contracted to provide an emergency ambulance service alongside the NAS. Each year these services respond to in excess of 300,000 ambulance calls ^32^. The National Emergency Operations Centre (NEOC) coordinates statutory emergency service responses to OHCA for most of the Irish state. In the Dublin metropolitan area EMS response is coordinated by both NEOC and the DFB east regional communications centre (ERCC). EMS care across the Irish state is provided by Paramedics or Advanced Paramedics who number approximately 2,500 & 700 respectively ^33^. All front-line ambulances must be staffed by at least a paramedic grade practitioner. A small number of EMS physicians supplement this care on a voluntary as available basis; however, they are not a core component of the statutory response.

Paramedic and Advanced paramedic scope of practice is determined by a statutory agency, the Pre-Hospital Emergency Care Council (PHECC). PHECC publish clinical practice guidelines and maintain a practitioner register ^34^. Only Advanced Paramedics are permitted to provide intravascular medications or to perform endotracheal intubation. Mechanical CPR devices are now widely available on all frontline ambulances. eCPR is generally not available in Ireland. In addition to statutory EMS providers, Ireland also has an extensive network of voluntary community first responders (CFRs) who can be dispatched by NEOC to OHCA to provide early CPR and defibrillation ^35, 36^.

### OHCA Health System Developments in Ireland 2012–2020

**Figure 1.**
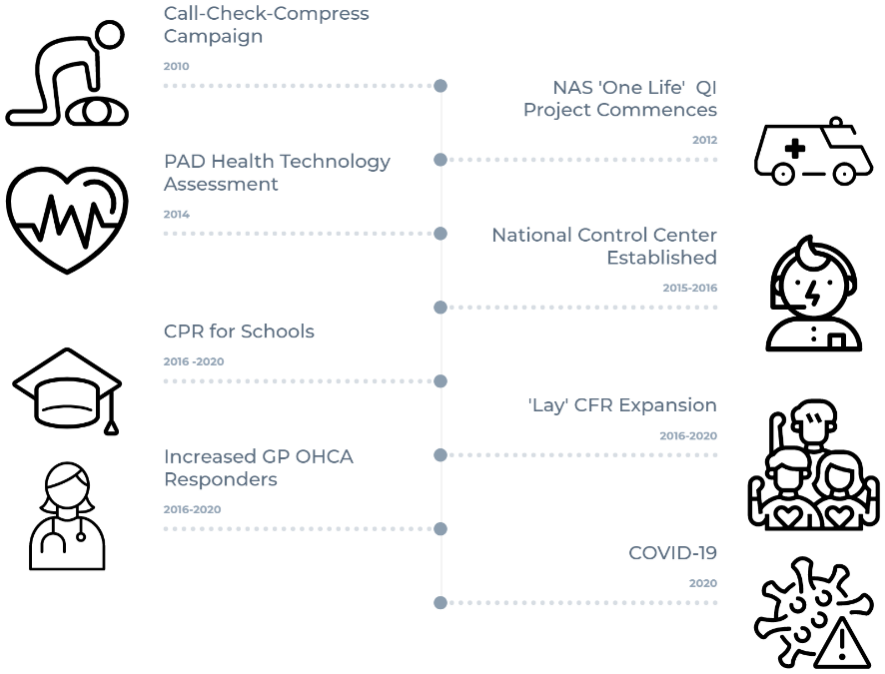
OHCA Health System Developments in Ireland 2012–2020. NAS: National Ambulance Service, QI: Quality Improvement, PAD: Public Access Defibrillation, CPR: Cardiopulmonary Resuscitation, CFR: Community First Responder, GP: General Practitioner, OHCA: Out-of-Hospital Cardiac Arrest.

Over the period 2012–2020 that OHCAR has been in existence, Ireland has undergone several health system developments that are pertinent to OHCA care. Figure 1 summarises a timeline of these developments. The Pre-Hospital Emergency Care Council national Citizen CPR or Call-Check-Compress programme was launched in 2010 ^37^. This public awareness campaign involved a series of national roadshows combined with a major national television, cinema, on-line and transport advertising designed to increase bystander CPR. Then in 2012 the NAS launched their ‘one life’ quality improvement programme ^38^. This ongoing programme focuses on several key aspects of OHCA including community interaction and public education, NEOC call taking and dispatch, EMS quality care on scene, and finally quality data management and audit processes. In 2014 the Irish Health Information and Quality Authority (HIQA) published a health technology assessment of public access defibrillation in Ireland ^39^. This was commissioned by government to inform decision making around proposed legislation to mandate public defibrillator availability. The health technology assessment estimated the clinical and cost effectiveness of a range of potential Irish public access defibrillation configurations, ranging from comprehensive to targeted ^39^. It estimated that between two and ten additional OHCA survivors could be achieved annually; however, none of the models achieved the threshold for cost effectiveness. It advised that targeted AED deployment in higher incidence locations in combination with an EMS-linked AED register and increased public awareness could potentially render the programmes’ cost effective ^39^. Ultimately the proposed legislation was abandoned, and a comprehensive national EMS-linked AED register is yet to be established.

 In 2015 and 2016 the National Ambulance Service transitioned from a system of multiple regional independent control centres to a single national control centre (NEOC). This significant change allowed an enhanced level of resource co-ordination and further allowed dispatch assisted CPR to be fully embedded as a standard of care at national level. Between 2016 and 2020 the Irish Heart Foundation ‘CPR 4 schools’ programme trained 1,827 teachers in 531 schools to perform CPR training for a potential 288,197 students. Between 2016 and 2020 lay community first responder groups increased from 100 to 175 nationally. In 2017 the NAS created four new Community Engagement Officer posts to support CFR activities and expansion. Also between 2016 and 2020 GP (general practitioner) first responder numbers doubled to almost 200 individuals ^40^. The final year of this study period 2020 is exceptional in that it represented the first year of the Covid-19 pandemic, the first wave of which occurred between February and July 2020 ^41^. The Irish health service responded by introducing a range of public health measures including travel restrictions, schools and business closure, cessation of large indoor gatherings, and other social distancing measures ^42^. In terms of OHCA, internationally Covid-19 is known to have detrimentally affected systems of care and was associated with prolonged EMS response and worse short-term outcomes including survival compared to pre-pandemic periods ^43^. The effects of COVID-19 on OHCA in Ireland have not yet been systematically evaluated; however similar trends are probably likely. Thus, it is necessary to consider this as a significant OHCA development in the 2020 period.

### Focus of this study

This study will examine data from the Irish Out-of-Hospital Cardiac Arrest Registry (OHCAR) to describe OHCA incidence and its care processes in Ireland over the period 2012 to 2020. We will further interrogate this data to identify key OHCAR variables that predict key outcomes. We anticipate approximately 18,000 relevant observations will be available for analysis. We will consider survival to hospital discharge, bystander CPR, and bystander defibrillation to be key outcomes of interest over the study period. We hypothesise that of the timeline of OHCA developments described above, two key developments are likely to be associated with a significant change in outcomes. Given the scale of reorganisation and centralisation of EMS control in 2015 and 2016 on the backdrop of the ongoing NAS ‘one life’ project we hypothesise that the NAS transition from a system of multiple regional independent control centres to the single national control centre (NEOC) would be associated with improvements in bystander CPR and survival to hospital discharge. In turn, given Covid-19’s detrimental effect on OHCA internationally we hypothesise that COVID-19 would be associated with significant reductions in bystander CPR, bystander defibrillation and survival to hospital discharge in Ireland.

### Study aims

The study will have four aims.

● To summarise key trends in available OHCAR data from the period 2012 – 2020
● To harness available OHCAR data to explore and model predictors of ‘bystander CPR’, ‘bystander defibrillation’ and ‘survival to hospital discharge’
● To explore the hypothesis that significant system level developments in 2015 & 2016 (the National Ambulance Service transition to a single national control centre) were associated with a temporal improvement in the above outcomes
● To explore the effect of COVID-19 on the above outcomes.

## Methods

### Study design

Key trends in available OHCAR data from the period 2012 – 2020 will be described and evaluated using descriptive summaries and graphical displays. Multivariable logistic regression will be used to model predictors of ‘bystander CPR’, ‘bystander defibrillation’ and ‘survival to hospital discharge’ and to explore the effects (if any) of system level developments in 2015/2016 and the COVID-19 pandemic on these outcomes. The R software for statistical computing will be used for analysing the data.

### Study population, setting and time frame

The population for this study will be patients of all ages who suffered un-witnessed, or bystander witnessed OHCA during the time period 2012 – 2020 and are included in the Irish national OHCAR cardiac arrest registry database. Patients who had an EMS witnessed OHCA will be specifically excluded from this current study. Follow on work will consider this distinct excluded group separately.

### Variables and categories of concern

All analysis will be based on the variables shown in Table 1. Variables 1–19 will be obtained from the OHCAR. Variables 20, 21 and 22 represent component time periods that are a priori considered to be potentially significant in the context of either system level developments or key population health challenges (Covid-19) during the study time period. Variables 20,21 and 22 will be dummy variables created using the ‘Year’ variable. Variables 20 and 21 will be linked dummy variables derived from the same 3-level categorical indicator of period and will be simultaneously included in regression models. We do not expect collinearity of these predictors, as transition and post-transition period correspond to years 2015 and 2016, and 2017 through 2020, respectively. The Covid period dummy variable (variable 22) will be an indicator of 2020 only, and thus should allow us to estimate any shift in survival in the final year of the study data. Table 1 highlights the independent variables that will be explored for each outcome of interest. Where an original OHCAR variable has multiple potential associated categories, we will collapse these categories to avoid decreased statistical power from analysis of an excessive number of potentially sparse categories. Original and collapsed categories are shown in Table 1. Season will represent the ‘winter’ healthcare period (October to March) as compared to other as ‘winter’ is a period of increased demand for the Irish healthcare service. The three time categories chosen are adopted based on previous OHCA research ^44^. Home (versus other) location was chosen as home represents the most common location for OHCA in Ireland. ‘Year’ (variable 9) will be treated as a continuous variable to conserve degrees of freedom and statistical power.

**Table 1.** Variables for analysis.

Variable Number	Variable	Categories	Collapsed Categories	Outcome of Interest		
				Bystander CPR	Bystander Defibrillation	Survival to Hospital Discharge
**1**	**Airway** ** Management**	**Basic ** **Management**	**None of the listed devices ** **used, OPA/NPA, No advanced** ** airway**			*
		**Supraglottic** ** airway device**				
		**Intubation**				
**2**	**Aetiology**	**Presumed Other**	**Trauma, Respiratory, ** **Submersion, Non-cardiac, ** **Other**	*	*	*
		**Presumed Cardiac**				
**3**	**Age**	**Age (years)**		*	*	*
**4**	**Sex**	**Female**		*	*	*
		**Male**				
**5**	**Call Response** ** Interval**	**Call Response** ** Interval (minutes)**		*	*	*
**6**	**Incident location**	**Other Location**	**Industrial, Public building, GP** ** Surgery, Farm, Sport place,** ** Residential institution, Street,** ** Ambulance, Other**	*	*	*
		**Home Location**				
**7**	**Mechanical CPR**	**No Mechanical ** **CPR Provided**				*
		**Mechanical CPR** ** Provided**				
**8**	**Season**	**Winter**	**October–March**	*	*	*
		**Other**	**April–September**			
**9**	**Year**	**Year (continuous** ** variable** ** 2012–2020)**		*	*	*
**10**	**First Shock ** **Delivered By**	**Not Applicable**				*
		**Bystander** ** Defibrillation**				
		**EMS Defibrillation**				
**11**	**Shockable Initial** ** Rhythm**	**Non-Shockable** ** Initial Rhythm**				*
		**Shockable Initial ** **Rhythm**				
**12**	**Chest ** **Compressions ** **Started By**	**EMS initiated CPR**				*
		**Bystander CPR**				
**13**	**Time of Day**	**Night**		*	*	*
		**Evening**				
		**Morning**				
**14**	**Total No of Shocks** ** Delivered**	**Total No of Shocks ** **Delivered**				*
**15**	**Who Witnessed** ** Collapse**	**Not Witnessed**		*	*	*
		**Bystander** ** Witnessed**				
**16**	**Urban or Rural**	**Rural Location**		*	*	*
		**Urban Location**				
**17**	**Weekday or ** **Weekend**	**Weekend**		*	*	*
		**Weekday**				
**18**	**Number of ** **Adrenaline Doses**	**Number of ** **Adrenaline Doses**				*
**19**	**Amiodarone** ** Administered**	**Amiodarone Not ** **Administered**				*
		**Amiodarone ** **Administered**				
**20**	**Transition Period** ** (2015 & 2016)**	**Transition Period ** **(2015 & 2016)**		*	*	*
		**Not Transition ** **Period**				
**21**	**Post Transition ** **Period (2017–2020)**	**Post Transition** ** Period ( 2017–2020)**		*	*	*
		**Not Post ** **Transition Period**				
**22**	**Covid Period**	**Covid Period (2020)**		*	*	*
		**Not Covid Period**				

### Statistical model building and interpretation procedures

For each outcome we will build a logistic model: initially a full model with all relevant predictors will be fitted. A refined model will then be built using a stepwise model selection procedure (STEPAIC function in R). This procedure builds several models from all possible combinations of the predictors by sequentially adding and dropping predictors and finally selects the model with the lowest AIC. The stepwise model will then be further improved by examining addition of pairwise interaction variables and retaining any interactions which improve fit. We will evaluate each model (full, stepwise and with interactions) based on AIC. In addition, the model deviance and a Hosmer-Lemeshow Goodness of Fit (GOF) test will be inspected for each model. To assess linearity between continuous predictors and the log odds of the outcome we will rely on good model fit as indicated by the Hosmer-Lemeshow goodness of fit test. We will also use logistic regression with higher order terms (polynomial terms) to explore if the relationship between the outcome and the continuous predictors is indeed non-linear. If higher-order terms are significant, these will be retained in the model, otherwise they will be dropped from the model. We will also attempt to ameliorate fit by including pair-wise interaction terms between predictors. We will summarise the effect of each individual variable in the final model using odds ratios and 95% confidence intervals. The effect of any significant interactions in the final model will be explored graphically. After selecting the final model from the three models, we will evaluate the predictive ability of the final best fitting model using 10-fold cross-validation and evaluate the prediction accuracy for the model.

### Missing data and sensitivity analysis

We anticipate some missing data in both the outcome and predictor variables. We will document the amount of missing data for each variable and graph missing data patterns across the entire dataset. To conduct sensitivity analysis, results from complete case analysis and multiple imputation will be presented. Multiple imputation will be done using the mice package in R, ten imputations will be derived, and the selected model will then be fit to these datasets, and the results will be compared with the complete case dataset.

## Ethical considerations

Research ethics approval has been obtained from National University of Ireland Galway, Research Ethics Committee (Reference 2020.01.012; Amend 2106).

The dataset used for this study will be anonymised prior to receipt by the research team. It will be impossible for the research team to identify or contact participants.

The National Ambulance Service Research Group have given permission for this anonymised data to be utilised for the purposes of this study.

OHCAR operates under ‘implied consent’. OHCAR does not contact patients, hospitals are advised to inform patients that they are included in OHCAR and what their rights in this regard are. Patients can have their data removed from the registry at their request.

## Dissemination plan

Study results will be disseminated via presentation at national and international scientific meetings and will be published in a peer reviewed scientific journal. No other associated data will be disseminated.

## Discussion

This project will provide the most comprehensive analysis of Irish out-of-hospital, cardiac arrest registry data to date. By exploring and modelling predictors of ‘bystander CPR’, ‘bystander defibrillation’ and ‘survival to hospital discharge’ over time, the project will yield a more granular and context specific understanding of the factors that can influence these key outcomes. In turn these data can be used to inform the evolution and future design of the system of community emergency care in Ireland. At the outset it is important to highlight that this project will have some important limitations. Beyond the OHCAR registry data set and the high-level overview of system developments presented in Figure 1, there is limited process data available on the system initiatives described. For instance, little data is available on community first responder activations over the relevant time period ^40^. Furthermore, the involvement of these responders in the OHCA care process has traditionally not been well captured although recent efforts will address this data deficit into the future ^36^. Ultimately if system developments are found to be associated with key outcomes it may be that in reality other confounding variables are in fact driving these outcomes. Previous work has demonstrated that the internationally agreed ‘Utstein’ registry variables that are the basis for this planned study explain only 51% of the variation in survival following OHCA ^45^. Thus, even following this planned research exercise important gaps in our understanding of OHCA outcomes in Ireland will remain. A further follow-on project is already planned to address this issue and is currently negotiating data linkage approvals. This project will aim to link OHCAR registry data with hospital in-patient data and geospatial census data to further enhance the scientific understanding of the variation in survival following OHCA. In the interim this current planned project serves to provide a critical baseline understanding of outcomes based on the registry dataset.

## Data Availability

No additional data are associated with this article.
