# Supplementary material for: Outcomes of out-of-hospital cardiac arrest in Ireland 2012-2020: Protocol for an observational study
Source: HRB Open Res. 2023 Oct 31;6:17. Originally published 2023 Mar 10. [Version 2] doi: 10.12688/hrbopenres.13699.2 (PMC10474347; doi:10.12688/hrbopenres.13699.2)
Supplement: Peer review history

# Reviewer report: Reviewer response for version 2

**DOI:** 10.21956/hrbopenres.15142.r36870

**Published:** 2023-11-10

**Authors:** Kyndaron Reinier

**Affiliations:** 1 Smidt Heart Institute, Cedars-Sinai Health System, Los Angeles, California, USA

The authors have adequately addressed concerns in my earlier review.

Is the study design appropriate for the research question?

Yes

Is the rationale for, and objectives of, the study clearly described?

Yes

Are sufficient details of the methods provided to allow replication by others?

Yes

Are the datasets clearly presented in a useable and accessible format?

Yes

Reviewer Expertise:

Prediction of out of hospital sudden cardiac arrest risk.

I confirm that I have read this submission and believe that I have an appropriate level of expertise to confirm that it is of an acceptable scientific standard.

# Reviewer report: Reviewer response for version 2

**DOI:** 10.21956/hrbopenres.15142.r36873

**Published:** 2023-11-08

**Authors:** Stuart Howell

**Affiliations:** 1 Monash University, Melbourne, Australia

I still have concerns over the assessment of effects associated with the transition period, the post transition period and the COVID period on the outcomes of interest. The authors don't expect collinearity because the 3 variables represent different time periods. However, this overlooks the fact that the time periods for the reference groups for these 3 variables overlap (Not transition, Not post transition and not COVID). The additional inclusion of year as a continuous variable in their models as implied in the variables section may exacerbate this.

 The analysis of the transition period versus not transition period (Q20) remains problematic since the reference category (not transition) would include 2012-2014 and 2017-2020 data. Any improvement post-transition could well be offset by poorer performance in the pre-transition period, resulting in estimates biased toward the null (no difference between transition and not transition).

Is the study design appropriate for the research question?

Partly

Is the rationale for, and objectives of, the study clearly described?

Yes

Are sufficient details of the methods provided to allow replication by others?

Yes

Are the datasets clearly presented in a useable and accessible format?

Not applicable

Reviewer Expertise:

Biostatistics; statistics; analysis of OHCA registry data.

I confirm that I have read this submission and believe that I have an appropriate level of expertise to confirm that it is of an acceptable scientific standard, however I have significant reservations, as outlined above.

# Reviewer report: Reviewer response for version 2

**DOI:** 10.21956/hrbopenres.15142.r36871

**Published:** 2023-11-02

**Authors:** Christopher M Smith

**Affiliations:** 1 Warwick Clinical Trials Unit, Warwick Medical School, University of Warwick, Coventry, England, UK

Thank you for clarifying the points raised last time.

Is the study design appropriate for the research question?

Yes

Is the rationale for, and objectives of, the study clearly described?

Yes

Are sufficient details of the methods provided to allow replication by others?

Yes

Are the datasets clearly presented in a useable and accessible format?

Not applicable

Reviewer Expertise:

Out-of-hospital cardiac arrest

I confirm that I have read this submission and believe that I have an appropriate level of expertise to confirm that it is of an acceptable scientific standard.

# Reviewer report: Reviewer response for version 1

**DOI:** 10.21956/hrbopenres.14984.r35455

**Published:** 2023-09-04

**Authors:** Kyndaron Reinier

**Affiliations:** 1 Smidt Heart Institute, Cedars-Sinai Health System, Los Angeles, California, USA

Summary:

 This manuscript provides a clearly written, comprehensive plan for analysis of the Irish Out of Hospital Cardiac Arrest Registry (OHCAR) data from 2012-2020. The authors have provided good rationale for their planned studies. They have also defined their research questions and outcomes explicitly, and carefully defined their variables of interest. The proposed analysis plan is appropriate.

Major comments:

1. In Figure 1, is the “Call-Check-Compress” campaign the same as the “Pre-Hospital Emergency Care Council national Citizen CPR” programme (described in text, pg. 4)? If so, consider clarifying this?
2. Table 1 – clarify in the text and in Table 1 whether the registry includes OHCA with EMS response but without attempted resuscitation (eg, an unwitnessed arrest with an EMS assessment that resuscitation would be futile, or an individual with EMS response but with a do-not-resuscitate order) and briefly describe how or if these cases will be included in analysis.
3. Table 1 – if the data is available, consider dividing non-shockable rhythm into pulseless electrical activity and asystole, at least for initial analyses.
4. Pg. 5, aims: For analysis of system-level developments (aim 3) consider excluding the COVID-19 pandemic time period, or if this time period is included, consider conducting a sensitivity analysis to determine whether conclusions are influenced by potential COVID-related changes in patient-level or system-level factors.

Minor comments:

1. Pg. 3, 2 ^nd^ para under heading of “Out of Hospital Cardiac Arrest & Quality Improvement Registries”, “Register (OHCAR)” should be “Registry (OHCAR)”.
2. Pg. 3, paragraph under heading of “Emergency medical services in Ireland”, the relationships between Health Services Executive and National Emergency Operations Center are a bit confusing.
3. Pg. 4, “potentially render the programmes’ cost effective” – the apostrophe is not necessary.
4. Figure 1 is very readable. However, I wonder whether it could be improved by putting community-level efforts (eg, Lay CFR expansion (?)) on one side and health system-specific efforts (eg, NAS One Life QI project) on the other? Please disregard if I have misinterpreted the distinction between the two efforts.

Is the study design appropriate for the research question?

Yes

Is the rationale for, and objectives of, the study clearly described?

Yes

Are sufficient details of the methods provided to allow replication by others?

Yes

Are the datasets clearly presented in a useable and accessible format?

Yes

Reviewer Expertise:

Prediction of out of hospital sudden cardiac arrest risk.

I confirm that I have read this submission and believe that I have an appropriate level of expertise to confirm that it is of an acceptable scientific standard, however I have significant reservations, as outlined above.

# Response

**Published:** 2023-10-27

**Authors:** Tomás Barry

Many thanks for your review and comments. Your input has been most useful in improving our paper.

- We have now clarified in the manuscript that the “Call-Check-Compress” campaign is the same as the “Pre-Hospital Emergency Care Council national Citizen CPR” programme.
- We have now clarified in the manuscript that during the period considered in this study OHCAR does not include OHCA with EMS response but without attempted resuscitation.
- We have not divided non shockable rhythm into PEA and asystole as this specific rhythm data is only available for a portion of cases. We anticipate though that follow on analysis will focus on patients with these individual rhythms for whom these data are available.

# Reviewer report: Reviewer response for version 1

**DOI:** 10.21956/hrbopenres.14984.r33549

**Published:** 2023-09-01

**Authors:** Christopher M Smith

**Affiliations:** 1 Warwick Clinical Trials Unit, Warwick Medical School, University of Warwick, Coventry, England, UK

Thank you for the opportunity to review this protocol. I have set out my thoughts and a few queries below.

**Rationale**

- The rationale for this project, the burden of OHCA and the potential to use this project as a springboard for inclusion is well described.
- Page 3, paragraph 5: “ *We anticipate that approximately fifty percent of cases recorded in OHCAR are witnessed (European average 66%), and twenty percent have an initial ‘shockable rhythm’ (European average 20%).*”
  Your reasoning for anticipating these figures is not clear to me. Is it from previous interrogation of the registry?
- There are a number of potential confounders that will affect the certainty of any conclusions drawn from this project. However, importantly, these limitations are recognised, have been explicitly stated in the discussion section, and there are plans to address these potential shortcomings in the future. This transparency and recognition of the limitation of registry-based research is important and good to see.

**Study Design**

- A review of data submitted to a national database, with a well-defined population. I have no concerns about the overall approach.

**Methods**

- I have some experience with logistic regression modelling, including when applied to an OHCA dataset. While the statistical methods sound reasonable to me, I must make it clear that I am not a statistical expert and cannot therefore provide a comprehensive statistical opinion or review.
- Page 7, paragraph 4: Ethical considerations: “ *hospitals are advised to inform patients that they are included in OHCAR and what their rights in this regard are.”*
  Do patients have the opportunity then to opt-out of having their data in the registry? I anticipate that this number of cases that this applies to would be small if so, but do you have any information about this?
- The Irish OHCAR is clearly the right source of information for this data. The registry and its case are adequately described. There is some potentially useful data that are not available from this source – e.g. actions of CFRs – but this limitation is acknowledged and discussed.

Some queries about the data being used:

1. Item 11: does non-shockable rhythm have PEA, asystole (+/- others e.g. bradycardias in infants) in its collapsed categories?
2. Page 6, Table 1, Item 20/21. I am slightly confused by this. If you were to look at item 20 alone, you might think that you were comparing both before (2012-2014) and after (2017-2020) together to the transition period (2015-16). I don’t quite understand that rationale as I think a reasonable hypothesis (alongside your existing hypothesis that the NEOC transition was a beneficial thing) might be that 2017-20 (or at least 2017-2019) outcomes would be better than 2012-14, as a result of the changes brought about during the transition phase. If you then look at item 21 I wonder whether you actually are doing a three-way comparison of before-during-after transition. This could perhaps be clarified.  If we then look at item 22, is there an argument for making the post-transition period 2017-19. If you have a strong argument for not doing that, then this could be made clearer.

**Other minor points**

- Last line of abstract, background: “ *and to explore the hypothesis that significant system level temporal developments were associated with improvements in these outcomes.*” This presumes that there were improvements in these outcomes in the first instance for you to hypothesise what the causes were. Would ‘changes’ rather than ‘improvements’ be a better word, or do you know already that these outcomes improved over the study time-period?
- Last sentence of abstract, discussion. You have capitalised and spelled out ‘Out-of-Hospital Cardiac Arrest’ rather than ‘OHCA’ used earlier.
- Page 3, paragraph 1: “appropriate” rather than “on appropriate”; “survive to hospital discharge” rather than “involve survival to hospital discharge”
- Page 3, paragraph 2: “ *CPR and defibrillation are complemented by several other OHCA interventions…*” Consider the wording of this, as you then go on to describe a number of interventions for which there is NOT (yet, perhaps) evidence of benefit at population level.  
- Page 3, paragraph 5: You use the term ‘Register’ not ‘Registry’ when describing the OHCAR.
- Page 4, last paragraph: “ *In 2015 and 2016 the National Ambulance Service transitioned from a system of multiple regional independent control centres to a single national control centre NEOC*”. Needs either a hyphen before or brackets around NEOC.
- Page 5, paragraph 2: “reduction” or similar may be a better word than “dis-improvement”.

Is the study design appropriate for the research question?

Yes

Is the rationale for, and objectives of, the study clearly described?

Yes

Are sufficient details of the methods provided to allow replication by others?

Yes

Are the datasets clearly presented in a useable and accessible format?

Not applicable

Reviewer Expertise:

Out-of-hospital cardiac arrest

I confirm that I have read this submission and believe that I have an appropriate level of expertise to confirm that it is of an acceptable scientific standard, however I have significant reservations, as outlined above.

# Response

**Published:** 2023-10-27

**Authors:** Tomás Barry

Many thanks for your review and comments. Your input has been most useful in improving our paper.

- Our anticipation that 50% of cases will be witnessed is indeed based on co-authors existing experience of the registry.
- We have clarified in the manuscript that patients can have their data removed from the registry at their request.
- Non-shockable rhythm does incorporate PEA ,asystole etc. For some patients the actual rhythm is documented, however for others it is only known whether the rhythm is shockable or non-shockable.
- In relation to the confusion concerning variables 20 and 21 we have now further clarified this in the manuscript. Our approach is to examine transition period and post-transition (including 2020 as the post-transition effect should still be present despite having a Covid-19 effect layered on top), as contrasts with pre-transition. Variables 20 and 21 are linked dummy variables, and will not be modelled separately.
- In relation to our hypothesis ‘that significant system level temporal developments were associated with improvements in these outcomes’, we appreciate your suggestion to use alternative language, however feel ‘improvements’ is appropriate as this is a hypothesis based on said temporal developments having a stated aim of improving those outcomes.
- Typographical suggestions have been incorporated as advised.

# Reviewer report: Reviewer response for version 1

**DOI:** 10.21956/hrbopenres.14984.r35454

**Published:** 2023-09-01

**Authors:** Stuart Howell

**Affiliations:** 1 Monash University, Melbourne, Australia

**Overall:**

 This is a unique study in that it is attempting to explore the impact of system level developments and COVID on trends in bystander CPR rates, bystander defibrillation and survival to hospital discharge as well as to identify key predictors of each outcome. I have no concerns with the overall design of the study as it replicates the methodology applied in similar research. However, I am not convinced that the proposed analysis will address the study’s aims. Some areas that the authors should consider are as follows:

**Study population:**

 The study population excludes EMS-witnessed arrests, which is reasonable and has precedent in previous research. The authors should also consider restricting the sample to arrests in adults as the characteristics of arrests in paediatric patients are fundamentally different to those in adult samples.

**Variable definitions:**

 The decision to restrict categorical variables to three levels seems arbitrary and the reclassification of variables should be determined on a variable-by-variable basis. There are two variables where this strategy leads to a loss of information – seasonality, which is dichotomised as winter versus summer, when a four-level categorical variable (spring, summer, autumn, winter) makes better sense; in time of day, afternoon is missing without any justification given apart from (presumably) the restriction to three categories.

 The authors should consider separating “Other” location (of arrest) into public places versus other (non-public) locations. Arrests in public locations are more likely to be witnessed, which may influence bystander CPR and defibrillation rates, and consequently, survival via these mechanisms.

 Age, year and epinephrine dose are included as continuous variables and non-linearity needs to be considered. In particular, non-linearity is implied with respect to year as your expectation is that the transition period will lead to improvements in the outcomes, while COVID will have a detrimental effect. The authors need to explain how they will assess and deal with any non-linearity in their continuous variables.

**Analysis**:

 I think that the authors’ proposed method for analysing the effect of the transition and COVID may lead to results that are biased. In variable 20, the transition period is compared to the non-transition period, which presumably includes the pre- and post- transition period. This may bias results to the null as gains in the post-transition period may be offset by poorer outcome performance in the pre-transition period. Similar problems may occur in aggregating the pre-transition and transition periods in the comparison with the post-transition period (variable 21) and when comparing the COVID to the pre-COVID period (variable 22). Furthermore, including all three variables as well as year may introduce problems with collinearity during modelling.

 In my opinion, the authors should consider a piecewise logistic regression model, which would provide them with separate slopes and intercepts to describe the trends associated with the pre-transition, transition, post-transition and COVID periods. They could then include the remaining predictors in their model (except for year, transition periods and COVID) which would describe the effect of each variable on the outcome after adjusting for the trend over periods. If the year range doesn’t provide sufficient data points for modelling, then the analysis could be done using quarter rather than year (i.e.: 36 quarters over the 9 year study period). It is easy enough to extrapolate the model coefficients to report the outcomes as annual trends if need be.

 The authors also need to be clear on what type of modelling they are undertaking. Their proposed approach is closely aligned to risk prediction (or prognostic) modelling where the goal is to develop an algorithm that allows the prediction of risk in an individual with a given set of covariate values. In this case, issues such as model discrimination and validity and prediction accuracy are important and should be investigated thoroughly.

 However, I’m not convinced that this consistent with the study aims. In my mind, the goal here is effect estimation, where the goal is to identify and describe variables that are associated with the outcomes of interest. In this instance, model reduction provides fewer gains than for risk prediction models and model validation is generally unnecessary.

 The authors should also note that stepwise regression has numerous problems and is no longer widely recommended as a variable selection strategy. Its application via automated programs such as STEPAIC is not to be recommended.

 The following book is a useful resource:

 Frank E Harrell, Jr. Regression modelling strategies. With applications to linear models, logistic and ordinal regression and survival analysis. 2 ^nd^ Edition. Springer Series in Statistics.

Is the study design appropriate for the research question?

Partly

Is the rationale for, and objectives of, the study clearly described?

Yes

Are sufficient details of the methods provided to allow replication by others?

Yes

Are the datasets clearly presented in a useable and accessible format?

Not applicable

Reviewer Expertise:

Biostatistics; statistics; analysis of OHCA registry data.

I confirm that I have read this submission and believe that I have an appropriate level of expertise to confirm that it is of an acceptable scientific standard, however I have significant reservations, as outlined above.

## References

[1] : Regression modelling strategies. With applications to linear models, logistic and ordinal regression and survival analysis. 2nd Edition. *Springer Series in Statistics* .

# Response

**Published:** 2023-10-27

**Authors:** Tomás Barry

Many thanks for your review and comments. Your input has been most useful in improving our paper.

- In relation to considering restricting the sample to arrests in adults. We agree that the characteristics of OHCA in paediatric patients may be different to those in adults. However, we also anticipate that even within the adult population there may be significant variation in characteristics. For this reason, we have adopted an initial whole population-based approach. We do acknowledge that follow on research that focuses on relevant subgroups may well be warranted.

- In relation to collapsing categorical variables we have clarified the justification in the manuscript as suggested on a variable by variable basis. Season represents the ‘winter’ healthcare period (October to March) v other as ‘winter’ represents a period of increased demand for the Irish healthcare service. The three time categories were adopted based on previous OHCA research ( https://doi.org/10.1016/j.ijcard.2020.09.043.) Home versus other location was chosen as home represents the most common location for OHCA in Ireland. While we agree that public locations are very important in terms of OHCA, some of the other categories do not clearly fall fully under public locations and thus a home v other categorisation was felt most appropriate. We do acknowledge that follow on research may be necessary to focus on OHCA at more specific locations.

- We acknowledge the reviewer's concern about potential non-linearity in our analysis, given the fact that we have several continuous predictor variables. In logistic regression models it is difficult to assess linearity between continuous predictors and the log odds of the outcome, so we rely on good model fit as indicated by the Hosmer-Lemeshow goodness of fit test. We will also use logistic regression with higher order terms (polynomial terms) to explore if the relationship between the outcome and the continuous predictors is indeed non-linear. If higher-order terms are significant, these will be retained in the model, otherwise they will be dropped from the model. We will also attempt to ameliorate fit by including pair-wise interaction terms between predictors.

- In relation to the proposed method for analysing the effect of the transition and COVID periods the reviewer is correct that an analysis of variable 20 alone would produce incorrect results. However, variables 20 and 21 are linked dummy variables derived from the same 3-level categorical indicator of period and will be simultaneously included in regression models. When variable 20 is adjusted for variable 21, the inference for transition period compared with pre-transition should be as expected. We do not expect collinearity of these predictors, as transition and post-transition period correspond to years 2015 and 2016, and 2017 through 2020, respectively. The Covid period dummy variable is an indicator of 2020 only, and thus should allow us to estimate any shift in survival in the final year of the study data.

- We appreciate your suggestion to consider a piecewise logistic regression model and note that this is similar to the approach we are taking with dummy-coding for period, along with the plan to test for pair-wise interactions (including between period dummy variables, and continuous year). The plan is to aggregate data by year, examining yearly trends, while also controlling for seasonal (winter v other) effects, and there are sufficient data points in each year to allow this approach.

- We do acknowledge the fact that our proposed methodology may be related to the prediction of risk or prognostic modelling. Our main goal in this study is, in fact, effect estimation. We aim to identify and describe variables that are associated with the outcomes of interest. Below are our considerations.

- Effect Estimation: Our main aim is to estimate the effects of various predictor variables on the binary outcomes. We want to understand how these factors are associated with the outcomes. We are not focused on developing a predictive algorithm for individual risk assessment.

- Model Reduction: We agree that for estimating effect sizes, extensive model reduction efforts may provide few gains and fail to reduce important confounding. We have some concerns about estimation with relatively rare interventions or small risk groups, in which context maintaining some precision can be done by removing predictors with no, or negligible, effects. We also view simple and easily interpretable models as an advantage.

- Model Validation: We appreciate the reviewer perspective that for effect estimation, model validation may be considered unnecessary. However, we still believe it is essential to assess the performance of our models, even in an effect estimation framework. Generation of predicted probabilities can provide diagnostics for outliers or poor fit for subsets of the sample, and re-sampling based methods also give a less biased estimate than the R-squared, for example, of the fit of the model, circumventing the ‘Winner’s Curse’ effect.

- In relation to the reviewers concerns regarding stepwise regression and the reference highlighted - We acknowledge that methods such as penalized regression have certain advantages for selecting variables with important effects, although may bias effect size towards zero. Given our concerns mentioned about inference with rare interventions or categories, and wishing to remove clearly irrelevant predictors, we believe the approach we have outlined is reasonable. We acknowledge that effect sizes may be inflated, and p-values too liberal, as is also the case in sample sizes this large (potentially including for clinically irrelevant effects). We do not plan to make claims of effects based on statistical significance, and will interpret the effect sizes and confidence intervals with a degree of caution given the inflation.

# Reviewer report: Reviewer response for version 1

**DOI:** 10.21956/hrbopenres.14984.r34169

**Published:** 2023-07-12

**Authors:** Marc Conterato

**Affiliations:** 1 North Memorial Emergency Medical Services, Robbinsdale, Minnesota, USA; 2 School of Medicine, University of Minnesota Twin Cities, Minneapolis, Minnesota, USA

This proposed study is well thought out and has a relatively broad scope to evaluate and discern the effect of multiple changes to the Irish OHCAR network and data capturing. The modeling proposed is quite well thought out and should yield a least two strongly linked initiatives that enhanced improvements in OHCAR. Use of the AIC system should yield adequate models to point to system changing events. 

 While some data may not be adequately captured do to information gathering deficiencies in the system, I would find it interesting if those were reported with the final study report, as they would indicate areas for further study and system improvement.

 In addition, it might be better for the study to possibly exclude or alternatively factor data that could be affected by COVID. It has been well shown that COVID caused an increase in cardiac arrest numbers and increase in mortality. My concern is that these data sets may produce confounding results that may affect the final modeling sets that are reached. Perhaps having the study show models with and without COVID data may offer clearer results.

Is the study design appropriate for the research question?

Yes

Is the rationale for, and objectives of, the study clearly described?

Yes

Are sufficient details of the methods provided to allow replication by others?

Yes

Are the datasets clearly presented in a useable and accessible format?

Yes

Reviewer Expertise:

Emergency medicine, EMS systems and oversight, pre-hospital cardiac resuscitation

I confirm that I have read this submission and believe that I have an appropriate level of expertise to confirm that it is of an acceptable scientific standard.

# Response

**Published:** 2023-10-27

**Authors:** Tomás Barry

Many thanks for your review and comments. Your input has been most useful in improving our paper.

- In relation to data not adequately captured, we endeavour to describe the level of missing data across the dataset and consider the implications when interpreting our findings.

- In relation to data that is potentially affected by COVID-19. We agree this is important and thus have included a dummy variable that represents the Covid period. This will allow us to model, estimate and adjust for any independent effect that may be present.
